# Protective effects of *Nippostrongylus brasiliensis-*derived uridine via the apical sodium-dependent bile acid transporter in a mouse model of TNBS-induced inflammatory bowel disease

**DOI:** 10.3389/fimmu.2025.1600838

**Published:** 2025-05-05

**Authors:** Caiyi Yuan, Qiang Wang, Yuying Chen, Xin Ding, Qiang Zhang, Jiakai Yao, Bei Zhang, Yang Dai, Hongxia Bai

**Affiliations:** ^1^ School of Public Health, Nanjing Medical University, Nanjing, China; ^2^ National Health Commission Key Laboratory of Parasitic Disease Control and Prevention, Jiangsu Provincial Key Laboratory on Parasitic and Vector Control Technology, Jiangsu Provincial Medical Key Laboratory, Jiangsu Institute of Parasitic Diseases, Wuxi, China; ^3^ Department of Parasitic Disease Prevention and Control, Guangzhou Tianhe District Center for Disease Control and Prevention, Guangzhou, China; ^4^ Department of Science and Education, Xishan People’s Hospital of Wuxi City, Wuxi, China

**Keywords:** Nippostrongylus brasiliensis, uridine, apical sodium-dependent bile acid transporter, inflammatory bowel disease, RNA sequencing

## Abstract

**Introduction:**

Inflammatory bowel disease (IBD), a chronic immune-mediated gastrointestinal disorder mainly covering Crohn's Disease and Ulcerative Colitis, has an unclear etiology. The exploration of novel intervention strategies remains a key scientific issue that is urgently needed for IBD treatment. The hygiene hypothesis has led researchers to notice that worm infections can regulate the immune system, which might help treat inflammatory diseases. *Nippostrongylus brasiliensis (Nb)*, similar to human hookworms in life cycle and symptoms, is often used in hookworm research. Our previous study also demonstrated that Nb-derived uridine screened from ES could exert anti-inflammatory and anti-atherosclerotic effects.

**Methods:**

In this study, we established the protective and anti-inflammation effect of Nb infection and ES intervention in TNBS-induced IBD model in mice and further validated the efficiency of uridine screened from ES. Moreover, we conducted an RNA sequencing (RNA-Seq) analysis to elucidate the relevant possible functional mechanisms responsible for the protective and anti-inflammation effects of ES or uridine administration.

**Results:**

Current results have demonstrated that uridine can exhibit a protective effect on TNBS-induced IBD in mice. Moreover, it was identified that *slc10a2* exhibited high expression after uridine intervention. By specific inhibition of the encoding protein (ASBT), its impact on the protective efficacy has been interrupted.

**Discussion:**

The current study has illustrated that uridine is capable of exerting potential therapeutic and anti-inflammatory effects on Inflammatory Bowel Disease (IBD) by modulating *slc10a2*. These findings could offer a novel therapeutic target for the intervention of IBD.

## 1 Introduction

Inflammatory bowel disease (IBD) is a chronic immune-mediated disorder of the gastrointestinal tract, mainly consisting of Crohn’s Disease and Ulcerative Colitis. From 1990 to 2017, the incidence rate of IBD rose from 3.7 million to nearly 6.8 million worldwide, causing a global burden of diseases (1). The incidence rate of IBD is still rising in the newly industrialized and developing countries and maintains a high stability in western countries (2). Currently, factors such as genetic susceptibility, host immune dysfunction, changes in gut microbiota, and environmental influences are considered to be the main pathogenesis of IBD (3). Benefit from the availability of multiple deep sequencing platforms, genetic research on IBD has also made significant progress that more than 200 genetic polymorphisms were identified to be linked with the increased risk of IBD such as adaptive immunity, mucosal immunity, and immune tolerance, and autophagy (4). The changes in the structure and function of gut microbiota could trigger host immune and metabolic responses which alter gut immune responses and ecosystems, exacerbating the symptoms of IBD (5). However, its exact etiology and specific drug are still need to be confirmed (6). The existing drug therapies only alleviate a few symptoms and are often accompanied by certain side effects, making it difficult to achieve a complete cure. Moreover, the recurrence of IBD seriously impairs patients’ quality of life and is highly correlated with the risk of colorectal cancer (7). Therefore, exploring novel intervention strategies is a crucial scientific issue urgently needed for IBD treatment.

In recent years, the “hygiene hypothesis” has provided new perspectives for pharmacological strategies in treating inflammatory diseases. This hypothesis posits that the development of immune system depends on its co-evolution with microorganisms. Excessively clean environments during childhood may lead to reduced exposure to microorganisms, affecting the normal development of the immune system and ultimately increasing the risk of autoimmune diseases in adulthood (8). Based on the hygiene hypothesis, the immune-regulatory functions of worms have been found to inhibit the progression of inflammation. This has been demonstrated in studies on worms therapies such as using schistosome and hookworm interventions to treat IBD, arthritis, celiac disease and other inflammatory conditions (9–11). Thus, the phenomenon of worm infection regulating the immune system has gradually attracted the attention of researchers in the search for treatment for certain inflammatory diseases.

Hookworms are common soil-transmitted nematode. The species that mainly infect humans include *Ancylostoma duodenale*, *Necator Americanus*, and *Ancylostoma ceylanicum.* Hookworm infections affect the health of approximately 500 million people in tropical regions worldwide, resulting in an annual loss of 3.2 million Disability Adjusted Life Years (DALYs), making it one of the most significant neglected tropical disease globally (12). Hookworms parasitize the human body, causing chronic infections and eliciting a type II immune response in the host. This response promotes B cells to secret IgE antibodies via the secretion of IL-4 and IL-13, initiating an anti-parasitic immune response that suppress the type I immune-related inflammatory response, minimizing host damage and facilitating tissue repair (12). Previous research has shown that experimental infection of *Necator Americanus* in humans can induce a Th2 immune response and inhibit the expression of IFN-γ and IL-17A, thereby alleviating inflammation in patients with celiac disease (13). A variety of worm-derived molecules, including proteins, lipids, and enzymes, have been identified as potentially exerting multiple immune-regulatory functions in different inflammatory disease models (14). Consequently, the iatrogenic “worm therapy” holds promise for treating diseases caused by immune system disorders, including IBD (15, 16), and opens up new avenues for the development of intervention methods for inflammatory diseases.

*Nippotrongylus brasiliensis* (*N*. *brasiliensis*, Nb) has a life cycle and pathogenic symptoms similar to those of human hookworms and is often used as an ideal model for hookworm research (17). Previous studies have found that Nb infection can trigger an immune response resembling the characteristics of type II response in human hookworm infections, including enhanced CD4+T cell dependent IgE production, eosinophilia, and mastocytosis (18). Relevant research has determined that NB-DNase II, a small molecule derived from Nb, could modulate innate immune responses and the development of dendritic cells, thereby activating regulatory T cells (Tregs) (19). Therefore, screening and analyzing molecules from Nb-derived products could be a novel strategy for devising therapeutic methods for inflammatory diseases. Our previous studies have screened out 10 active molecules with anti-inflammatory effects from 45 metabolites of ES (excretory and secretory products) (20). ES, being the main product of adult Nb parasitism in the host intestine, has been found to inhibit ovalbumin-induced mouse asthma (21). In our previous study, we used ultra-performance liquid chromatography-mass spectrometry (UHPLC-MS) to perform untargeted metabolomics analysis on the intestinal contents of mice infected with Nb and ES from Nb-derived products. We found that the content of uridine increased significantly compared to the normal control (20). Uridine is an essential pyrimidine nucleoside for RNA synthesis and participates in several crucial metabolic processes (22). According to relevant research reports, uridine has shown certain protective effects in multiple animal models of inflammatory diseases (23, 24). Additionally, our previous study also demonstrated that Nb-derived uridine screened from ES can exert anti-inflammatory and anti-atherosclerotic effects *in vitro* and *in vivo* against atherosclerosis (25). Therefore, hookworm infection can regulate the host’s immune response to effectively mitigate the progression of autoimmune diseases, presenting a new approach for the development of IBD interventions based on hookworms.However, it is still necessary to verify whether IBD, as a classic inflammatory disease, can be intervened by ES or uridine.

In this study, we established the protective and anti-inflammation effect of Nb infection and ES intervention in a TNBS-induced IBD mouse model and further validated the efficacy of uridine screened from ES. We also conducted an RNA sequencing (RNA-Seq) analysis to identify differentially expressed genes in mice intervened with ES or uridine. Based on these findings, we elucidated the possible functional mechanisms responsible for the protective and anti-inflammation effects of ES or uridine administration in IBD mice, aiming to provide therapeutic targets for the immunotherapy of IBD.

## 2 Materials and methods

### 2.1 Animals, parasites and ethical statement

Sprague Dawley rats (male, 300 g) and C57BL/6 mice (8 weeks old, female) were provided by the Animal Center of Jiangsu Institute of Parasitic Diseases (JIPD, Wuxi, China). The Nb was donated by Professor Alex Loukas at James Cook University and has been stored and maintained in our laboratory for approximately seven years. All rats and mice were acclimated to the feeding environment for one week before being used in the experiment. Normal feeding was maintained during the experiment to ensure a favorable living environment for the animals. The Ethical Committee of the JIPD approved all experimental work involving animals (accession numbers: JIPD-2020-007 and JIPD-2021-001). All animals used in this study were housed in a standard environment with a temperature ranging from 21°C to 25°C, a humidity level of 60% to 65%, a 10 to 14-hour light–dark cycle, and unrestricted access to food and water.

### 2.2 Nb culture and ES preparation

Approximately 3500 L3 stage larvae were subcutaneously injected into rats. Rat feces were collected after infection until the 9th day. A mixture of 5 μg/mL amphotericin solution, carbon powder, and corn cob padding was added to the feces, which was then transferred to the absorbent paper in a culture dish for cultivation at 26°C. After 1 week, larvae were observed migrating to the edge of the culture dish. These larvae were collected and washed in PBS. The infection process was repeated. On day 7 post-infection, adult worms were collected from the small intestines of infected rats and used for ES production according to our previous method (20, 25). The suspension was transferred to a sterile 50-mL centrifuge tube and washed twice with Dulbecco’s phosphate-buffered saline (DPBS) containing antibiotic antimycotic (Gibco-Thermo Fisher, Waltham, MA, USA) and then transferred into a flat bottom 24-well plate and cultured for 7 days in RPMI-1640 medium (Gibco-Thermo Fisher) at 37°C and 5% CO_2_. The culture supernatant was collected and replaced with fresh medium every 24 hours. After centrifugation at 2,000 g and filtration through a 0.22 mm filter to remove eggs, parasite fragments and remaining solution, the final excretory-secretory products (ES) were obtained and stored at −80 °C for later use.

### 2.3 IBD modeling and protective efficacy evaluation by Nb and its derived products

**Figure 1:**
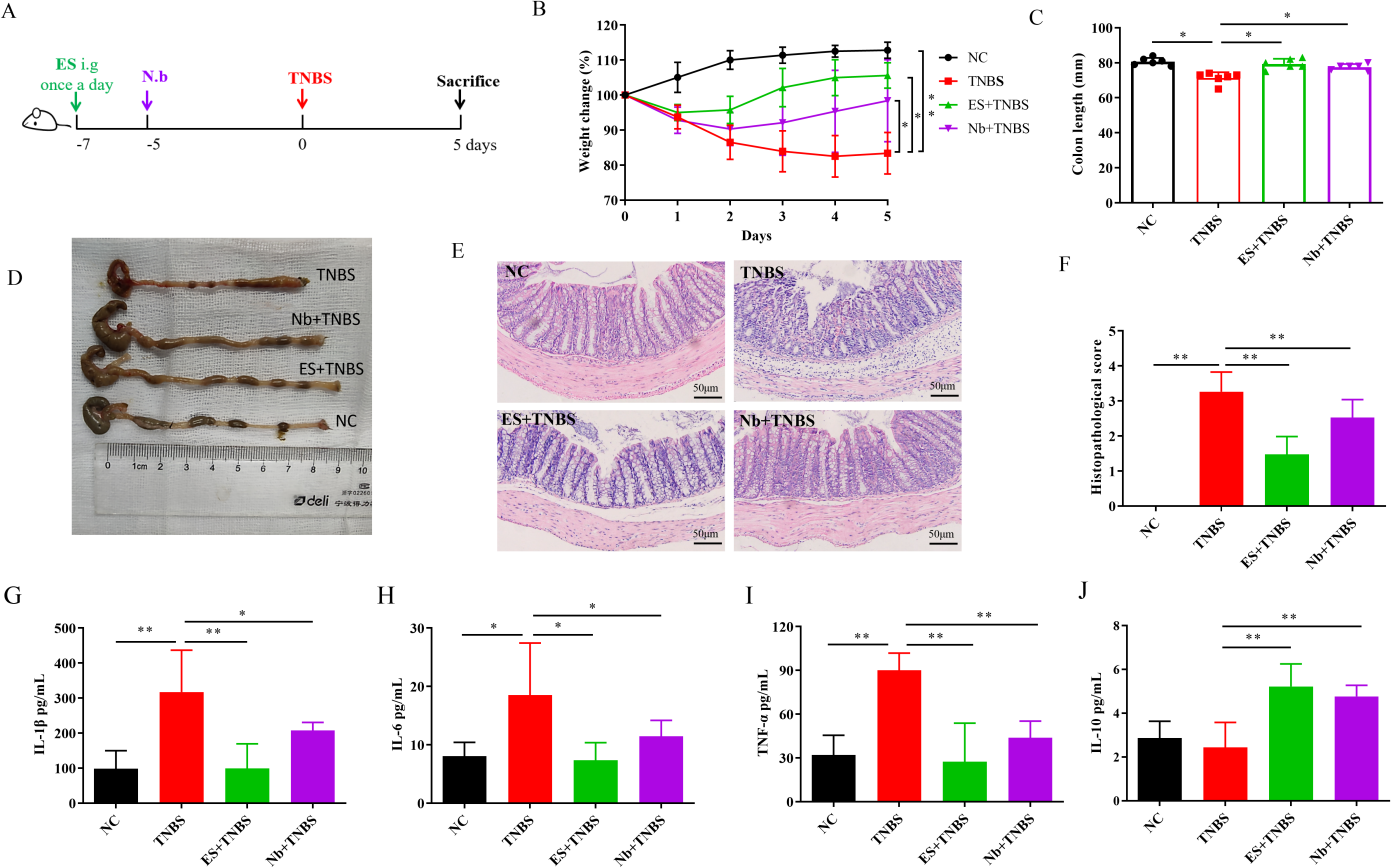
Nb infection and ES intervention confer protection against TNBS-induced IBD in mice (n=6). **(A)** Schematic illustration of the experimental design of Nb and ES administration in IBD model. **(B)** Changes in body weight over 5 days in comparison with the modeling day. **(C, D)** Comparative and statistical analysis of colon length. **(E)** Transverse section of the mouse colon stained with HE (under 400× magnification). **(F)** Histological score of colon. **(G–J)** Levels of inflammatory-related cytokines in the colon following Nb infection and ES intervention in mice including IL-1β, IL-6, TNF-α and IL-10 (n = 6). * and ** means *p* < 0.05 and 0.01, respectively.

Due to its advantages of easy induction and good reproducibility, 2, 4, 6-Trinito-benzene-sulfonic acid (TNBS) is widely used in the study of the pathogenesis of IBD and the screening of potential therapeutic drugs, as it possesses similar immunological and histopathological features to human IBD (26). A total of 24 C57BL/6 mice were randomly divided into four groups, including normal control group (NC group), TNBS modeling group (TNBS group), Nb infection in TNBS model (Nb + TNBS group), and ES intervention in TNBS model (ES + TNBS group), with 6 mice in each group for validating Nb and ES in IBD. Mice in the ES group were orally administered 200 μL of ES daily for 7 days before modeling, while mice in the Nb group were subcutaneously injected with 500 Nb L3 stage larvae on day 5 before modeling. Except for the NC group, mice in other groups were perfused with a 40 mg/mL TNBS (Sigma-Aldrich, MO, USA) solution through the rectum (100 μL per mouse) for IBD modeling on day 0. Body weight was recorded daily, and the mice were euthanized on the other 5^th^ day. The colon was separated and removed for subsequent measurement (**Figure 1A**).

**Figure 2:**
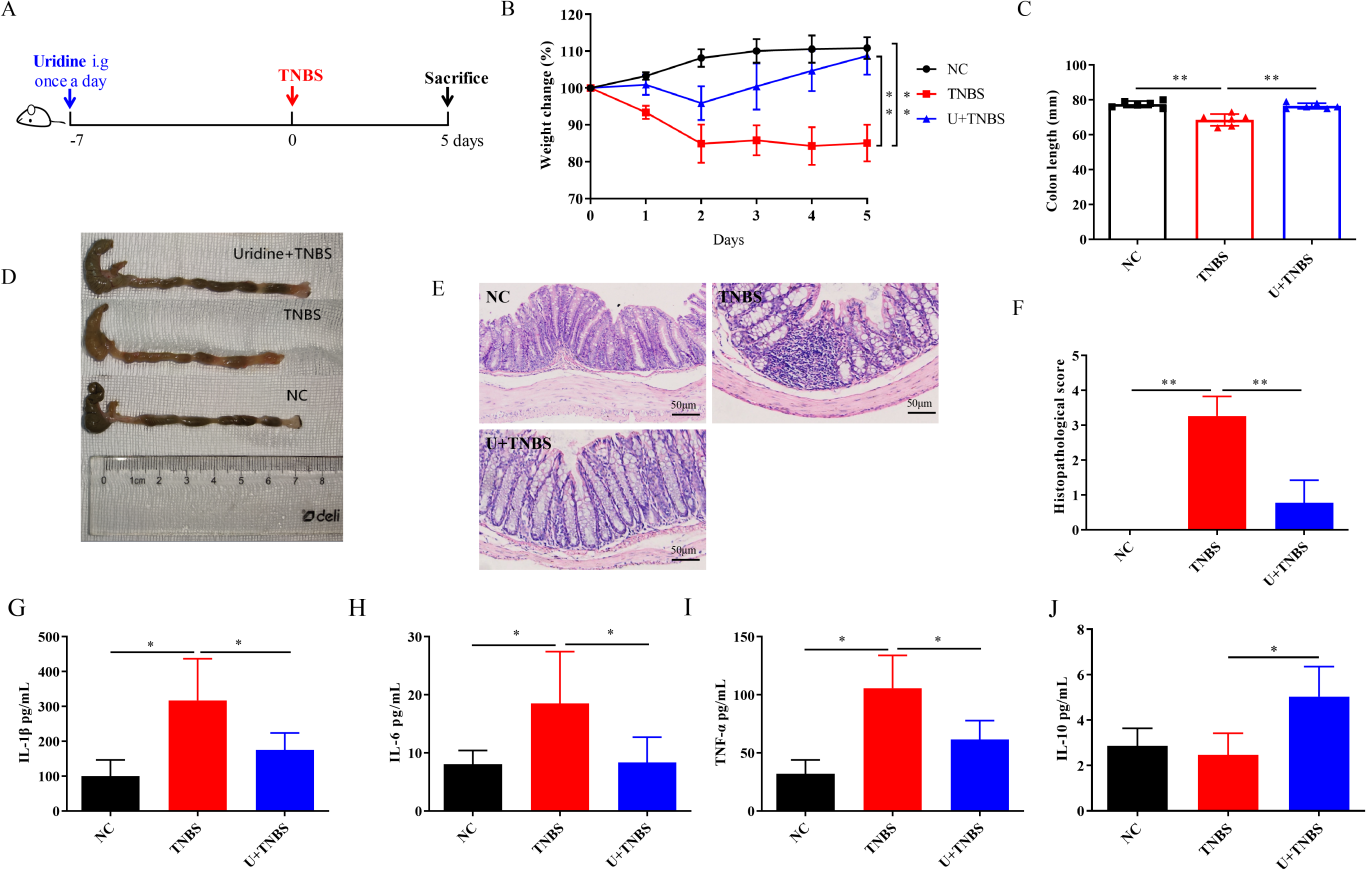
Uridine intervention confers protection against TNBS-induced IBD in mice (n=6). **(A)** Schematic illustration of the experimental design of uridine administration in IBD model. **(B)** Changes in body weight over 5 days in comparison with the modeling day. **(C, D)** Comparative and statistical analysis of colon length. **(E)** Transverse section of the mouse colon stained with HE (under 400× magnification). **(F)** Histological score of colon. **(G–J)** Levels of inflammatory-related cytokines in the colon following uridine intervention in mice including IL-1β, IL-6, TNF-α and IL-10 (n = 6). * and ** means *p* < 0.05 and 0.01, respectively.

For Nb derived uridine assessment experiment, an additional 18 C57BL/6 mice were randomly allocated into three groups (with 6 mice in each group), including the NC group, TNBS group, and uridine intervention in TNBS model (U + TNBS group). Mice in U + TNBS group were orally gavaged with 10 mg/kg of uridine on a daily basis for 7 days prior to the modeling process. On day 0, mice in both the TNBS group and Uridine group underwent colitis induction with 100 μL of a 40 mg/mL TNBS solution by rectal enema. The body weight and activity of each mouse were monitored daily starting from the commencement of the experiment. On the 5^th^ day after TNBS administration, all mice were euthanized and the colon was removed for subsequent measurement (**Figure 2A**).

### 2.4 Inflammatory-related cytokines detection

For the detection of cytokine levels detection, approximately 1 cm from the bottom of the colon was excised, washed with PBS and homogenized in PBS on ice. Then, an equal volume of RIPA buffer containing protease inhibitors was added, and the tissue was lysed at room temperature for 30 minutes with gently agitated throughout the lysis process. After centrifugation to remove sediment, the total protein concentration of supernatant from colonic tissue was quantified by BCA assay (Absin, Shanghai, China). The detection of cytokines in the mouse colon was carried out using TNF-α/IL-1β/IL-6 (Absin, Shangai, China) and IL-10 ((Solarbio, Beijing, China) ELISA kits according to the instructions (intra- and inter-assay CVs were less than 10%).

### 2.5 Histological analysis

After euthanizing all the mice, the colon was dissected and washed with PBS to remove the feces. Approximately 1 cm of the distal colon was collected and then immersed in 4% paraformaldehyde overnight for fixation. The colon tissue was sectioned using a semiautomatic microtome (RM2235, Leica, Wetzlar, Germany) and stained with hematoxylin-eosin (HE) solution after being embedded in paraffin. The tissue sections were observed under a microscope (BX63, Olympus, Tokyo, Japan) and scored according to the following grading criteria: 0, no inflammatory; 1, slight leukocyte infiltration, with visible infiltration less than 10% under high magnification and no structural changes; 2, moderate leukocyte infiltration, with the infiltration area being 10% to 25%, crypt elongation, and the thickening of the intestinal wall not exceeding the mucosal layer and no ulcers; 3, high leukocyte infiltration, with the infiltration area being 25% to 50%, crypt elongation, thickening of intestinal wall, and appearance of superficial ulcers; 4, diffuse leukocyte infiltration, with the infiltration area exceed 50%, crypt elongation and curvature, thickening of intestinal wall, and occurrence of extensive ulcers.

### 2.6 RNA sequencing and RT-qPCR analysis for colon tissues

Colon samples of mice were randomly selected from the TNBS group, ES+TNBS group and Uridine +TNBS group for RNA sequencing (RNA-Seq) analysis. Firstly, RNA was extracted and then subjected to agarose gel electrophoresis and a microplate reader for quantitative quality inspection to determine the purity and integrity of RNA samples. Secondly, mRNA was enriched using magnetic beads with Oligo dT (Thermo Fisher Scientific, MA, USA) and then randomly fragmented by fragmentation buffer (Agilent Technologies, CA, USA). Using the above mRNA as a template, the first strand of cDNA was synthesized by random hexamers primers (Thermo Fisher), followed by the addition of buffer, dNTPs, and DNA polymerase I (Qiagen, Germany) to synthesize the second strand of cDNA. Finally, the double stranded cDNA was purified via AMPure XP beads (Beckman Coulter, CA, USA). The purified double stranded cDNA was subjected to terminal repair, followed by the addition of an A tail and ligation of sequencing adapters. Then, fragment size selection was performed using AMPure XP beads, and finally PCR enrichment was performed to construct a cDNA library. The constructed library was sequenced using the Illumina HiSeq™ platform (Illumina, CA, USA) to obtain raw reads, and subsequent bioinformatics analysis was based on clean reads, which were obtained by removing reads containing adapters, low-quality, and uncertain base from raw reads. Clean reads were aligned with the reference genome through HISAT2 software, and the FPKM values of gene expression in each sample were calculated via feature Counts software. Finally, differentially expressed genes from ES+TNBS group and Uridine +TNBS group were screened based on the corrected *p* value (< 0.05) and log2foldchange (> 1) when compared to that in the TNBS group. Additionally, a comparative analysis between the differentially expression genes from ES+TNBS group and Uridine+TNBS group was conducted. Further, the overlapping genes were selected for KEGG signaling pathway enrichment analysis to determine the most significantly top 20 among them.

For further confirmation of the potential target genes (obtained above) for uridine, another 1 cm colon samples from all groups were collected and stored at −80°C for further mRNA expression levels detection by reverse transcription-PCR (RT-PCR) using Applied Biosystems QuantStudio7 Pro (Thermo Fisher). Primers are shown in **Table 1**. RNA was extracted by the FastPure Complex Tissue/Cell Total RNA Isolation Kit (Vazyme, Nanjing, China), and its concentration was measured via NanoDrop2000 (Gibco-Thermo Fisher). Reverse transcription was performed according to the HiScript IV All-in-One Ultra RT SuperMix for qPCR (Vazyme, Nanjing, China) and the reaction system was operated by HiScript IV All-in-One Ultra RT SuperMix for qPCR (Vazyme, Nanjing, China). The reaction conditions contained pre-denaturation (95°C for 300 s) and 40 denaturation cycles (95°C for 10 s, 60°C for 30 s) and dissolution curve (95°C 15 s, 60°C 60 s, 95°C 15 s).

**Table 1:** Primers used in present study.

Genes	Primers (5’-3’)
*slc10a2-*F	TAGATGGCGACATGGACCTCA
*slc10a2-*R	CCCGAGTCAACCCACATCTTG
*β-actin-*F	TGACGTTGACATCCGTAAAGACC
*β-actin-*R	CTCAGGAGGAGCAATGATCTTGA

### 2.7 Mechanism exploration for uridine in alleviating IBD

As observed in the RNA sequencing and RT-qPCR analysis above, the gene, *slc10a*, was selected as a potential target of uridine, which encodes Apical Sodium-Dependent Bile Acid Transporter (ASBT) and can be inhibited by Linerixibat (GSK2330672). To explore the *in vivo* mechanism of uridine’s action, the group intervened with both uridine and GSK2330672 in TNBS model (U + TNBS + GSK group) was added in this part. Mice in this group were orally gavaged with 100 μL of GSK2330672 at a concentration of 10 mg/kg twice a day for 7 days (27). The experimental procedures for the other groups remained unchanged (**Figure 4A**). Cytokine detection and histological analysis were also conducted following the methods described above in sections 2.4 and 2.5.

### 2.8 Statistically analysis

The statistical analysis was performed using IBM SPSS 22 Statistics (IBM Corporation, Armonk, NY, USA). One-way ANOVA and independence *t*-test were employed to compare means and determine statistical differences between different conditions. *p* values were denoted as * *p* < 0.05 and ** *p* < 0.01 to indicate significant differences.

## 3 Results

### 3.1 Protective effects of Nb infection and ES intervention against TNBS-induced IBD

Compared with the NC group, mice in the TNBS group exhibited a significant reduction in body weight after modeling. Both Nb infection and ES intervention effectively mitigate the weight loss induced by TNBS in the IBD model (**Figure 1B**, p < 0.05). The colon length in the TNBS group was significantly shorter than that in the NC group, however, the shortening was less pronounced in both the Nb + TNBS and ES + TNBS group (**Figures 1C, D**, p < 0.05). In contrast to the NC group, TNBS treatment caused severe pathological damage, including structural destruction of colonic crypts, loss of goblet cells, and significant inflammatory infiltration. In the Nb + TNBS group and ES + TNBS group, the epithelial layer and crypt structure were clearly visible, with reduced infiltration (**Figure 1E**) resulting in a significant increase in the pathological score (**Figure 1F**, p < 0.01).

The levels of pro-inflammatory cytokines, such as IL-1β, IL-6, and TNF-α, in the colon tissue of the TNBS group, were significantly higher than those in the NC group (**Figures 1G, I**, p < 0.05 or < 0.01). After Nb infection or ES intervention, the levels of those pro-inflammatory cytokines were significantly reduced compared to the TNBS group (**Figures 1G, I**, p < 0.05 or < 0.01). The level of anti-inflammatory cytokine IL-10 was significantly increased in the Nb + TNBS and ES + TNBS group compared to the TNBS group, while no significant changes were observed in the TNBS group compared to the NC group (**Figure 1J**, p < 0.01). These results indicated that Nb infection and ES intervention could alleviate the progression of IBD by inhibiting the inflammatory responses in TNBS-induced IBD mouse model.

### 3.2 Protective effects of Nb-derived uridine against TNBS-induced IBD

**Figure 3:**
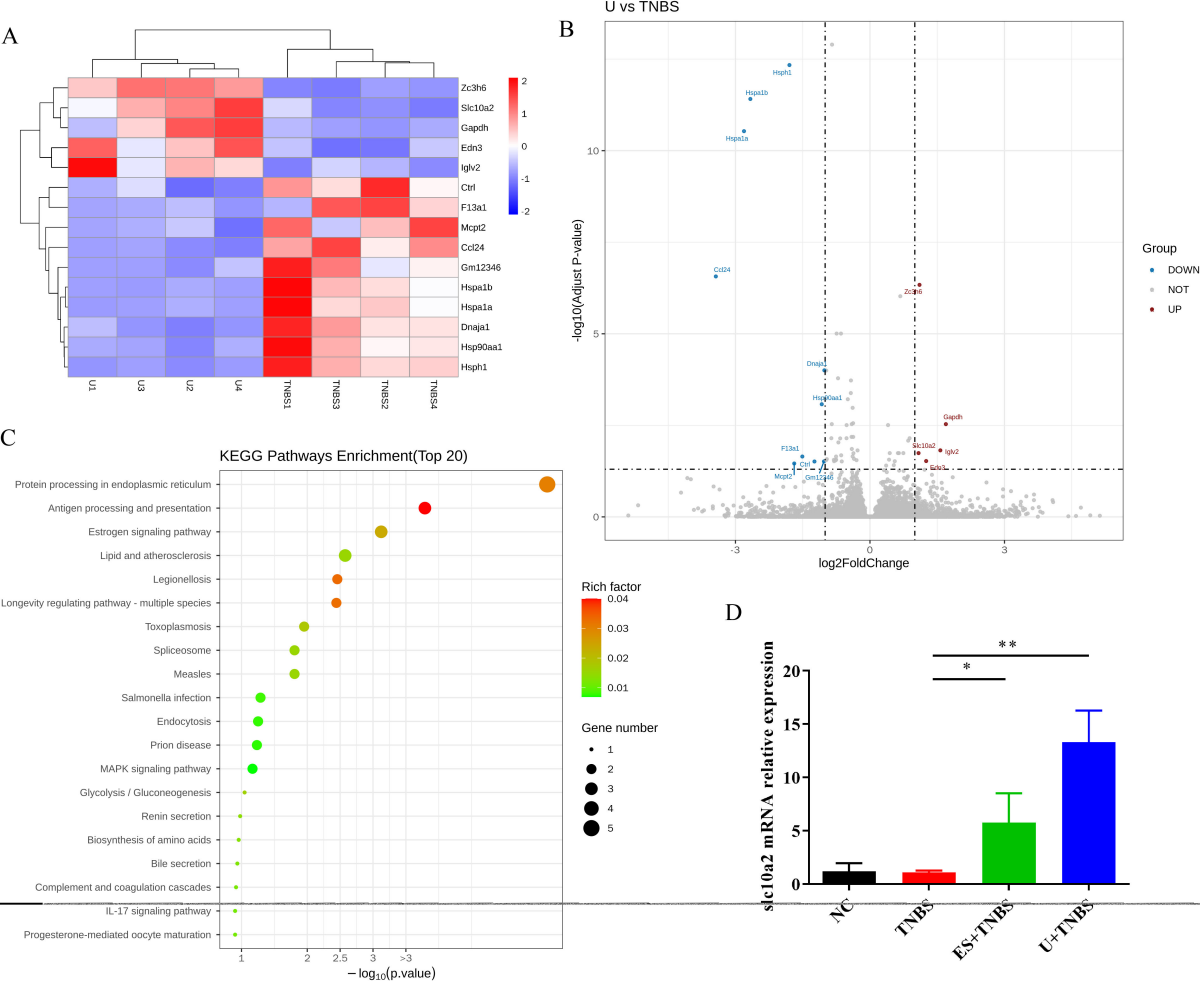
RNA-Seq analysis of colon tissue between TNBS and uridine group. **(A)** Cluster diagram of differentially expressed genes between TNBS and uridine group (*n* ≥ 3). **(B)** Volcanic map of differential gene expression distribution. **(C)** KEGG enrichment bubble plot of differentially expressed genes. **(D)** Colon mRNA relative expression of *slc10a2* by RT-qPCR (*n* ≥ 6).

As previously reported, uridine, a metabolite from adult Nb, is the major component of ES products and has demonstrated good anti-inflammatory activity both *in vitro* and *in vivo* (21). However, it protective effect on TNBS-induced IBD remains to be further explored. As shown in **Figure 2**, uridine intervention effectively alleviate the weight loss caused by TNBS-induced IBD in mice (**Figure 2B**, *p* < 0.01). The colon length of the TNBS group was significantly shorter than that in the NC group, and severe edema and bleeding were observed in the colon tissue (**Figures 3C, D**, *p* < 0.01). The degree of colon injury in the U + TNBS group was significantly improved compared to that in the TNBS group (**Figures 2C, D**, p < 0.01). Compared with the TNBS group, the U + TNBS group exhibited less pathological damage with a more intact colonic epithelial layer and crypt structure (**Figures 2E, F**, p < 0.01).

Regarding the levels of inflammatory-related cytokines in colon tissues, uridine intervention reduce the upregulation of pro-inflammatory cytokine levels caused by TNBS induced IBD, including IL-1β, IL-6, and TNF-α (**Figures 2G, I**, p < 0.05), and promoted the expression of IL-10 in mice colon (**Figure 2J**, p < 0.05). These results indicated that uridine could also exert a protective effect against TNBS induced IBD in mice by reducing the pro-inflammatory response and enhancing anti-inflammatory efficiency.

### 3.3 Association between slc10a2 expression and uridine’s protective effect against TNBS-induced IBD

To explore the possible mechanism underlying uridine’s protective effect against IBD, RNA-Seq technology was used to screen and identify downstream target genes. In this study, four colon samples from the TNBS group, ES+TNBS group and U+ TNBS group were selected for RNA-Seq analysis. Compared with the TNBS group, the distribution of differentially expressed genes in U + TNBS group was visualized using a heat map and a volcano plot. A total of 15 differentially expressed genes were identified, among which *slc10a2*, *zc3h6*, *gapdh*, *edn3*, and *iglv2* were significantly upregulated (**Figures 3A, B**).

Based on the analysis of differentially expressed genes, KEGG signaling pathway enrichment analysis was performed to determine the top 20 most significantly enriched signaling pathways. The results showed that the differentially expressed genes in the U + TNBS group were mainly enriched in antigen processing and presentation, protein processing in the endoplasmic reticulum, MAPK signaling pathway, bile secretion, estrogen signaling pathway, and IL-17 signaling pathway (**Figure 3C**). Furthermore, a comparative analysis between the differentially expression genes from ES+TNBS group and U+TNBS group was conducted, only two genes, *slc10a* and *edn3*, were upregulated in the both sets of comparisons (**Figure 3A**; **Supplementary Figures S1, S2**). A more in-depth investigation showed that *slc10a2* encodes the Apical Sodium-dependent Bile Acid Transporter (ASBT), which is closely associated with the bile acids transportation (28). The bile acid signaling pathway plays a pivotal role in regulating intestinal immunity and the microbial flora, making it a promising therapeutic target for IBD (29). The gene expression level of *slc10a2* was further verified by using RT-qPCR. It was significantly upregulated after ES and uridine interventions compared to the TNBS group (**Figure 3D**, p < 0.05 and < 0.01, respectively). Based on this evidence, it was speculated that the expression of *slc10a2* might be associated with the protective effect of uridine against TNBS-induced IBD.

### 3.4 Importance of apical sodium dependent bile acid transporter in uridine’s protective effect against TNBS-induced IBD

**Figure 4:**
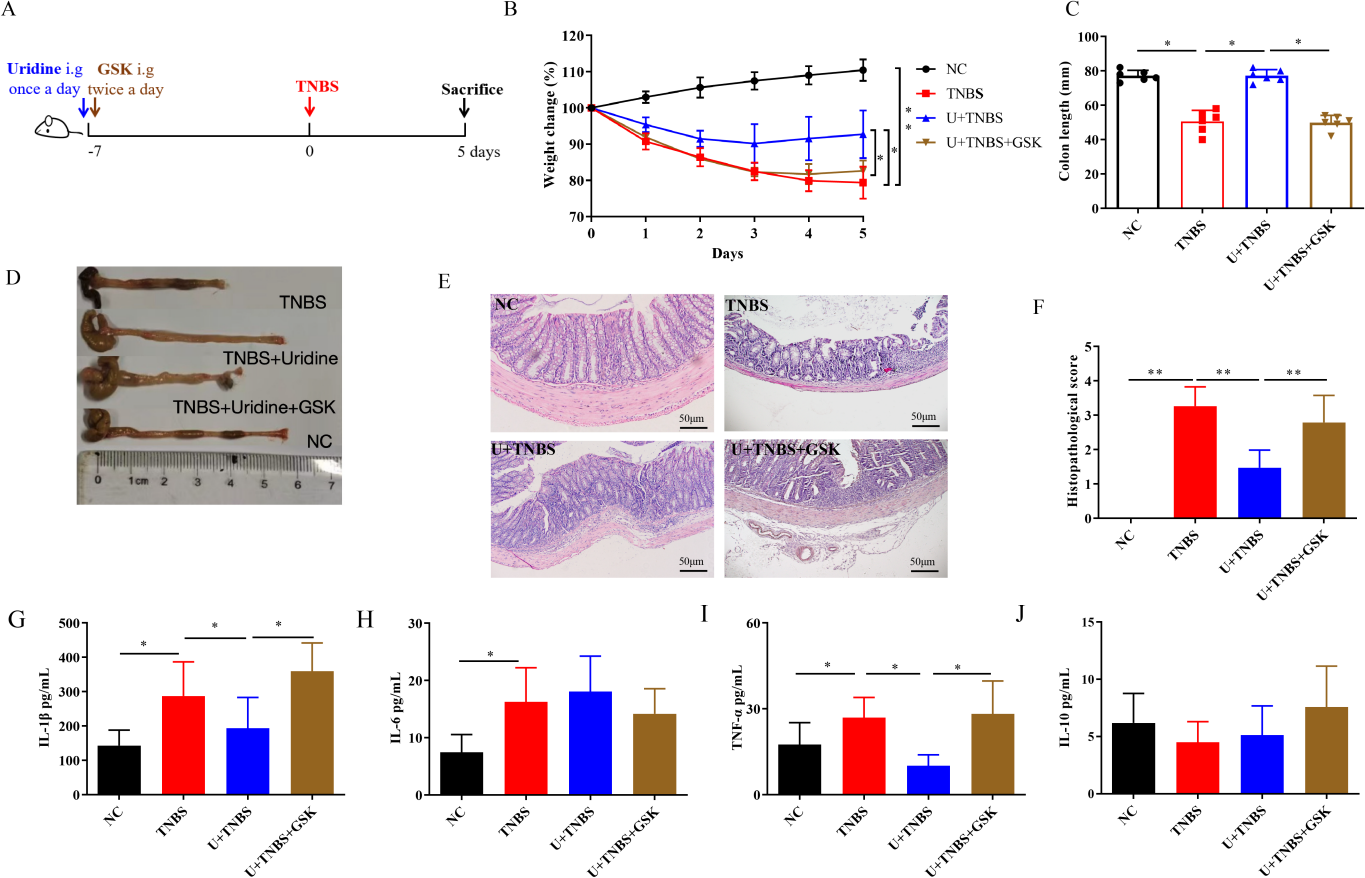
The inhibition of *slc10a2* attenuated the protective effect of uridine against TNBS-induced IBD in mice (n=6). **(A)** Schematic illustration of the experimental design in IBD model. **(B)** Changes in body weight over 5 days in comparison with the modeling day. **(C, D)** Comparative and statistical analysis of colon length. **(E)** Transverse section of the mouse colon stained with HE (under 400× magnification). **(F)** Histological score of colon. **(G–J)** Levels of inflammatory-related cytokines in the colon following uridine and GSK intervention in mice including IL-1β, IL-6, TNF-α and IL-10 (n=6). * and ** means *p* < 0.05 and 0.01, respectively.

As mentioned above, *slc10a2* encodes ASBT, which can be inhibited by Linerixibat (GSK2330672). To further investigate the role of *slc10a2* expression in uridine’s protective effect against TNBS-induced IBD, an animal experiment was conducted. The results showed that, compared with the Uridine + TNBS group, the addition of inhibitor intervention failed to alleviate intestinal damage and weight loss (**Figures 4B, C**, p < 0.05). Moreover, that addition of inhibitor intervention maintained the intestinal impair caused by TNBS (**Figure 4D**, p < 0.05). Histological findings indicate that, compared to the Uridine + TNBS group, the addition of the inhibitor intervention did not reduce histological damage; instead, it led to a higher pathological score (**Figures 4E, F**, p < 0.01). Additionally, the addition of inhibitor intervention to the Uridine + TNBS group upregulated the levels of IL-1β and TNF-α (**Figures 4G, I**, *p* < 0.05), while no significant changes were observed in levels of IL-6 and IL-10 (**Figures 4H, J**). Collectively, these results suggest that specific inhibition of ASBT can counteract the anti-inflammatory effect and the protective effect of uridine against TNBS-induced IBD.

## 4 Discussion

The small molecules present in the excrement and secretions of adult hookworms parasitizing in the host’s small intestine play a crucial role in the host-pathogen interaction. Previous metabolomics studies have revealed that non-protein small molecule metabolites derived from Nb possess anti-inflammatory bioactivity, which makes them worthy of further exploration as a novel strategic approach for treating inflammatory diseases (30, 31). This study demonstrated that uridine, a highly expressed metabolite screened from ES products, can protect against TNBS-induced IBD in mice (21). Through RNA sequencing analysis of the colons, it was found that *slc10a2* was highly expression after uridine intervention. By specifically inhibiting the encoded protein, ASBT, and observing its impact on the protective efficacy, the underlying mechanism of uridine’s protection in IBD was further investigated. We speculated that ASBT could be an effective therapeutic target for uridine to achieve anti-inflammatory and protective functions in TNBS-induced IBD. However, further experimental verification is needed in the future.

The TNBS-induced colitis model exhibits classic immunological and histopathological characteristics reminiscent of human IBD, and is widely used of IBD pathogenesis and the screening of potential therapeutic agents (26). This model typically causes symptoms such as diarrhea, reduced food intake, and weight loss in mice (26). Inflammatory bowel disease (IBD) and many other inflammatory disorders are characterized by elevated expression of pro-inflammatory cytokines, such as TNF-α, IL-6 and IL-1β (32). The increased secretion of those pro-inflammatory cytokines can disrupt the tight junction barrier in the intestinal epithelium, leading to increase epithelial permeability and exacerbating the inflammatory cascade. Conversely, IL-10 plays a crucial role in suppressing intestinal mucosal damage and inflammation (33, 34). In this study, both Nb infection and ES intervention effectively mitigated the histopathological lesions, body weight decline, and reduction in pro-inflammatory cytokines levels induced by IBD in mice. Given the metabolomic analysis of uridine in ES, we further evaluated the protective efficacy of ES intervention and the correlation between its component uridine in the subsequent experiments.

Uridine is essential for maintaining cellular function and energy metabolism, and has been widely used to alleviate cell toxicity and enhance neurophysiological function (35, 36). It has been shown to have anti-inflammatory effects in various experimental animal models, such as those of asthma, pneumonia, and arthritis. These effects are achieved by inhibiting the secretion of pro-inflammatory cytokines and relieving related symptoms (23, 24, 37). Our previous research screened uridine from the excretory and secreted products of Nb and further investigated its therapeutic effects on inflammatory diseases like atherosclerosis (25). Uridine was found to have anti-inflammatory and anti-atherosclerotic effects both *in vitro* and *in vivo*, making it a key metabolite in ES products (25). In this study, we found that the prophylactic administration of uridine, screened from ES, in the IBD mouse model significantly ameliorate the progression of IBD and reduced damage to the colon tissue. The uridine-treated group showed a decrease in the expression of pro-inflammatory factors and an increase in anti-inflammatory factors. These results were consistent with those from another dextran sulfate sodium (DSS)-induced colitis model, in which colonic instillation of uridine effectively protected against inflammation by regulating inflammatory cytokines in local tissues and circulation (38). Collectively, these studies suggest that uridine, as a small molecule selected from ES products, can be considered the main active molecule in ES for the treatment of IBD in mice.

High-throughput RNA sequencing can comprehensively obtain the complete information of all mRNA in the required tissues and organs under specific conditions, and is a novel technique for studying gene transcription and regulatory characteristics. In this study, based on a portion of the RNA-seq data, it was hypothesized that the therapeutic effect of uridine on Inflammatory Bowel Disease (IBD) might be related to several upregulated genes. Among them, *slc10a2* was identified, which is closely related to bile acid transport (28). Through the bile acid signaling pathway, the Apical Sodium-Dependent Bile Acid Transporter (ASBT) encoded by *slc10a2* can inhibit inflammation, stimulate the production of anti-inflammatory factors, and promote the regeneration of the epithelial barrier, making it a potential therapeutic target for IBD (29). Some other studies have also shown that the therapeutic effect of glucocorticoids in IBD can be partially attributed to the increased expression of ASBT (39). To verify the potential molecular mechanism of uridine’s therapeutic effect on IBD, specific and irreversible blockade of ASBT was carried out. With the addition of the inhibitor, uridine intervention failed to alleviate the pathological damage in enteritis, and no significant difference was detected in the expression of pro-inflammatory cytokines compared with the TNBS group. These results indicated that blocking ASBT could lead to the failure of uridine to protect against the pathological damage of enteritis and control inflammation.

There were some limitations in this study. ES, as the main product from Nb, is a complex composed of soluble proteins, small molecules, and extracellular vesicles (20). Although uridine could exert similar anti-inflammatory and protective effects in this study, it cannot fully represent the full efficacy of ES that further research is still needed on other components. Meanwhile, uridine itself has certain pharmacological effects that further verification is also necessary to determine the probable synergistic effects of other components together in ES.

## 5 Conclusion

In conclusion, this study demonstrated that uridine exerts potential therapeutic and anti-inflammatory effects on Inflammatory Bowel Disease (IBD) through the modulation of *slc10a2*. These findings not only deepen our understanding of IBD pathogenesis but also provide a novel therapeutic target for future IBD interventions. However, further studies are warranted to elucidate the detailed molecular mechanisms of uridine-mediated *slc10a2* regulation and to evaluate its therapeutic efficacy and safety in clinical settings.

## Data Availability

The datasets presented in this study can be found in online repositories. The names of the repository/repositories and accession number(s) can be found in the article/**Supplementary Material**.

## Supplementary material

Supplementary Figure S1 Cluster diagram of differentially expressed genes between TNBS and ES group (*n* ≥ 3).

Supplementary Figure S2 RNA-Seq analysis of colon tissue between TNBS and ES group. **(A)** Volcanic map of differential gene expression distribution. **(B)** KEGG enrichment bubble plot of differentially expressed genes. (*n* ≥ 3).
