# Supplementary figures and images for: Protective effects of Nippostrongylus brasiliensis-derived uridine via the apical sodium-dependent bile acid transporter in a mouse model of TNBS-induced inflammatory bowel disease
Source: Front Immunol. 2025 May 5;16:1600838. doi: 10.3389/fimmu.2025.1600838 (PMC12087013; doi:10.3389/fimmu.2025.1600838)

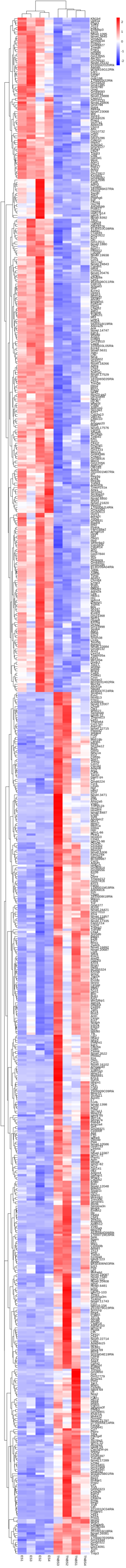

Supplement: Supplementary Figure S1 — Cluster diagram of differentially expressed genes between TNBS and ES group (n ≥ 3). [file Image1.png]

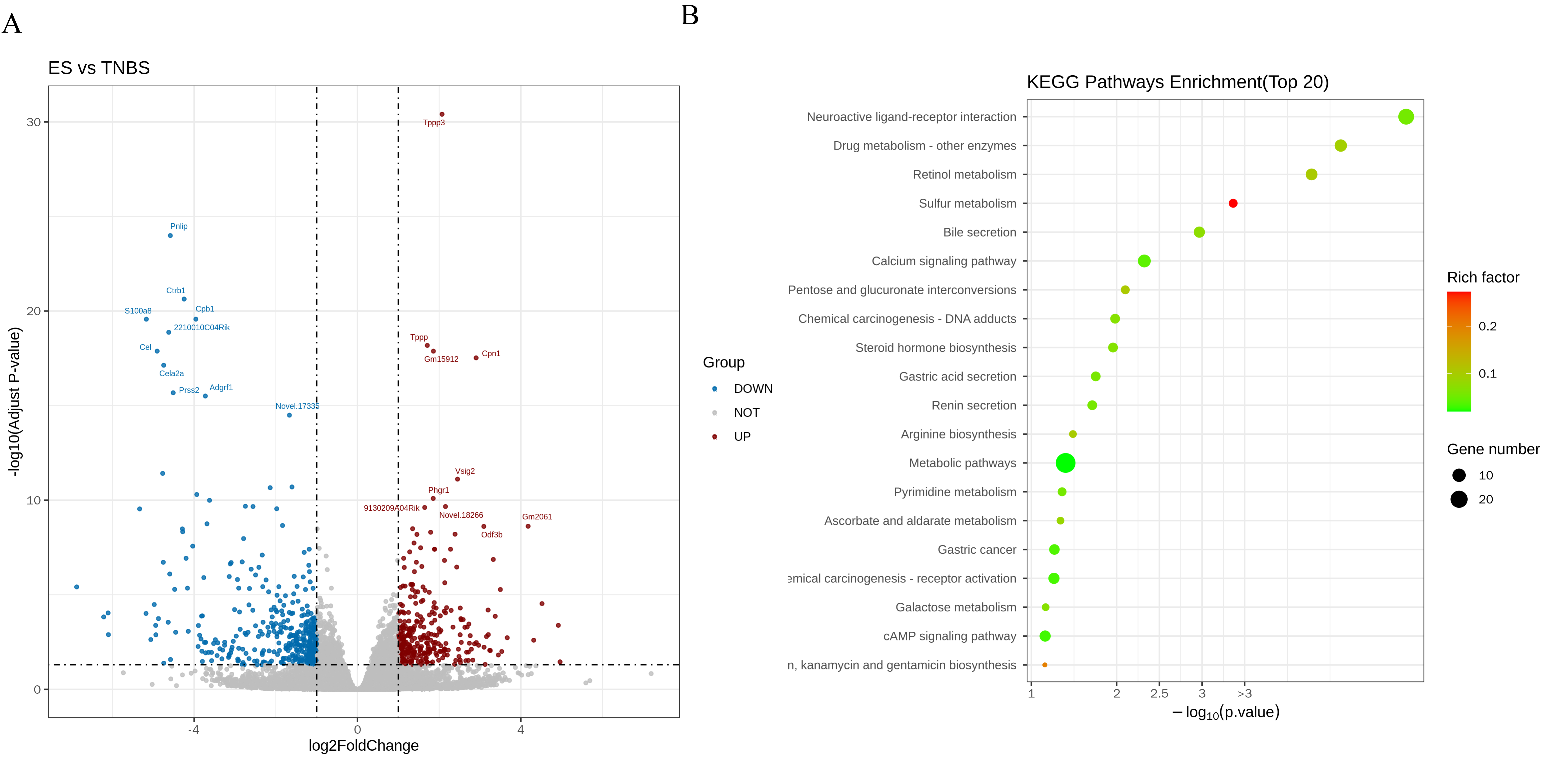

Supplement: Supplementary Figure S2 — RNA-Seq analysis of colon tissue between TNBS and ES group. (A) Volcanic map of differential gene expression distribution. (B) KEGG enrichment bubble plot of differentially expressed genes. (n ≥ 3). [file Image2.tif]
